# Comparison of two sperm processing techniques for low complexity assisted fertilization: sperm washing followed by swim-up and discontinuous density gradient centrifugation

**DOI:** 10.5935/1518-0557.20160040

**Published:** 2016

**Authors:** Cássio L Fácio, Lígia F Previato, Ligiane A Machado-Paula, Paulo CS Matheus, Edilberto Araújo Filho

**Affiliations:** 1 Center of Human Reproduction of São Jose do Rio Preto - São José do Rio Preto - SP, Brazil

**Keywords:** Intrauterine insemination, semen processing, swim-up, discontinuous concentration gradient

## Abstract

**Objective:**

This study aimed to assess and compare sperm motility, concentration, and morphology recovery rates, before and after processing through sperm washing followed by swim-up or discontinuous density gradient centrifugation in normospermic individuals.

**Methods:**

Fifty-eight semen samples were used in double intrauterine insemination procedures; 17 samples (group 1) were prepared with sperm washing followed by swim-up, and 41 (group 2) by discontinuous density gradient centrifugation. This prospective non-randomized study assessed seminal parameters before and after semen processing. A dependent t-test was used for the same technique to analyze seminal parameters before and after semen processing; an independent t-test was used to compare the results before and after processing for both techniques.

**Results:**

The two techniques produced decreases in sample concentration (sperm washing followed by swim-up: *P*<0.000006; discontinuous density gradient centrifugation: *P*=0.008457) and increases in motility and normal morphology sperm rates after processing. The difference in sperm motility between the two techniques was not statistically significant. Sperm washing followed by swim-up had better morphology recovery rates than discontinuous density gradient centrifugation (*P*=0.0095); and the density gradient group had better concentration recovery rates than the swim-up group (*P*=0.0027).

**Conclusion:**

The two methods successfully recovered the minimum sperm values needed to perform intrauterine insemination. Sperm washing followed by swim-up is indicated for semen with high sperm concentration and better morphology recovery rates. Discontinuous density gradient centrifugation produced improved concentration recovery rates.

## INTRODUCTION

Spermatozoa undergo a series of biochemical and structural changes within the female genital tract called capacitation (Neves, 1991). In-vivo, capacitation occurs over a period of seven hours (Yoshida *et al*., 2008). During this period, the glycoprotein coat and seminal proteins are removed from the surface of the sperm's acrosome. Capacitated spermatozoa show highly active flagellar beating (hyperactivation), undergo the acrosome reaction, penetrate the pellucid zone, and finally bind and fuse with the oocytes (Yoshida *et al*., 2008).

Sperm capacitation in assisted human reproduction (AHR) is performed artificially using specific techniques. Intrauterine insemination (IUI), an assisted reproduction technology (ART), is a non-invasive method and the least expensive of ARTs (Dodson & Haney, 1991). Several factors affect the success rates of IUI; severe male, tubal, and peritoneal factors and severe endometriosis had to be excluded (Scemama *et al*. 1995). Seminal parameters also significantly impact the outcome of IUI (Arny & Quagliarello, 1987; Brasch *et al*. 1994; Berg *et al*., 1997; Dorjpurev *et al*., 2011).

Sperm processing techniques for IUI vary from laboratory to laboratory and even from patient to patient (Zhao *et al*., 2004). Furthermore, these techniques have become very useful in the treatment of male infertility, allowing for better sperm recovery and improvements in sperm motility rates (Barroso, *et al*., 2005).

The purpose of semen processing is to increase the concentration of motile sperm, and to remove seminal plasma, debris, prostaglandins, and other substances deleterious for sperm viability that cause uterine contractions and bacterial contamination (Pasqualotto, 2007). Removal of immotile sperm, leukocytes, or immature germ cells is another advantage of these techniques, and one that can be an important factor for increasing seminal quality (Aitken & Clarkson, 1987). Therefore, sperm used in IUI must be processed (i.e., separated) from the seminal fluid, capacitated, and then selected based on morphology and motility to be able to be introduced into the uterine cavity (Huang *et al*., 1996).

The swim-up technique is easy to perform, cost-effective, and usually recovers a very clean fraction of highly motile spermatozoa (Henkel & Schill, 2003).

Sperm processing by the discontinuous concentration gradient technique usually recovers a clean fraction of highly motile spermatozoa, thus allowing the separation of spermatozoa from ejaculates with very low sperm density and providing good yield, largely eliminating leukocytes and significantly reducing reactive oxygen species (Henkel & Schill, 2003).

This study aimed to assess and compare sperm motility, concentration, and morphology recovery rates, before and after processing through sperm washing followed by swim-up or discontinuous density gradient centrifugation in normospermic individuals.

## MATERIAL AND METHODS

Fifty-eight semen samples from 17 couples submitted to double homologous IUI cycles from 2008 to 2010 in the Center for Human Reproduction of São José do Rio Preto, SP, Brazil, were included in this prospective non-randomized study. Some patients underwent IUI in more than one reproductive cycle.

The individuals enrolled in the study met the following inclusion criteria: male patients were normozoospermic; female patients were aged 38 years or younger, had mild endometriosis, and cervical factor infertility with at least one normal fallopian tube as determined by hysterosalpingography or video laparoscopy. Exclusion criteria included severe endometriosis, tubal-peritoneal factor, and male factor infertility. Enrolled couples signed informed consent terms before joining the study.

The 58 semen samples were divided into two groups: group 1 with 17 samples submitted to sperm washing followed by swim-up; and group 2, with 41 samples submitted to discontinuous density gradient centrifugation.

Semen parameters (concentration, motility, and morphology) were used in the choice of technique. Samples with sperm concentrations below 20 million cells/ml, sperm motility equal to or greater than 50%, and patients with previous failed IUI procedures were excluded from the swim-up group and included in the discontinuous density gradient centrifugation group.

### Ovarian stimulation protocol

All patients underwent ovarian stimulation with 100 mg/day of clomiphene citrate from days 3 to 7 of the menstrual cycle, along with subcutaneous human menopausal gonadotropin (hMG, 75 IU) (Menopur; Ferring Ltda, Brazil) at days 3, 5, and 7 of the cycle.

Transvaginal ultrasound (baseline) (Midray-Expert 3C5A; China) was performed on day 2 of the cycle for all patients before ovarian hyperstimulation. Follicular development was monitored by transvaginal ultrasound starting on day 8 or 9 of the cycle and later depending on each case; and when at least one follicle reached 20 mm in diameter, 5000 IU of human chorionic gonadotropin (hCG) (Ovidrel, Serono, Brazil) were administered subcutaneously. IUI was scheduled 36 to 40 hours after hCG once ovulation was confirmed.

### Semen analysis and capacitation

Semen samples were collected by masturbation after 3-5 days of sexual abstinence. The seminal parameters were analyzed by only one observer and categorized according to the 1999 WHO criteria (WHO, 1999): complete liquefaction within 60 minutes; sperm concentration (M/ml) ≥ 20 million; motility (grade A+B) > 50%; morphology ≥ 14%.

Seventeen semen samples from group 1 were processed through sperm washing followed by swim-up. First, the sample was washed with modified human tubal fluid (mHTF) medium with HEPES (Irvine Scientific), supplemented with 15% synthetic serum substitute (SSS, Irvine Scientific), and centrifuged at 270 × g for 5 minutes (Excelsa Baby I - Model 206, FANEN, São Paulo, Brazil); the supernatant was subsequently removed. After that, the swim-up technique was performed, in which the pellet was resuspended and homogenized in modified human tubal fluid (mHTF) medium with HEPES until a volume of 1 ml was reached. The resuspended pellet was removed with a Pasteur pipette and added into another 15 ml conical Falcon tube containing 1 ml of modified human tubal fluid (mHTF) medium with HEPES (care was taken so that both did not mix). The tube was inclined at an angle of 45o and incubated for 50 minutes at 37oC. Then, 0.5 ml was removed from the surface of the supernatant to perform post-processing seminal parameter analysis.

Forty-one semen samples from group 2 were processed using the discontinuous density gradient centrifugation method using the Isolate stock (Irvine Scientific, Santa Ana, CA, USA). The total volume of semen was divided so that a 15-ml conical Falcon tube contained 1 ml of a 90% density lower layer, 1 ml of a 50% density upper layer, and 1 ml of semen (1/1/1). The sample was then centrifuged at 270 × g for 15 minutes (Excelsa Baby I - Model 206, FANEN, São Paulo, Brazil).

After centrifugation, the supernatant was removed and the spermatozoa (pellet) was placed into another 15-ml Falcon tube, which contained 5 ml of modified human tubal fluid (mHTF) medium with HEPES (Irvine Scientific), supplemented with 15% synthetic serum substitute (SSS, Irvine Scientific); it was then centrifuged at 270 × g for 10 minutes. The final pellet was resuspended in the same medium solution, obtaining a final volume of 1 ml. A 10-µl aliquot was used to perform the analysis of post-processing seminal parameters.

### Statistical analysis

A dependent t-test was performed for group 1 samples comparing semen parameters before and after semen processing by sperm washing followed by the swim-up technique; for group 2, the comparison involved semen parameters before and after semen processing by the discontinuous density gradient centrifugation technique.

An independent t-test was performed to compare the seminal parameters of samples in groups 1 and 2 at two different times: (i) before semen processing and (ii) after semen processing.

Data analysis was carried out using the SAS 9.3 System for Windows; differences yielding p-values of 5% or lower were deemed statistically significant.

### RESULTS

The assessment of each technique in terms of semen parameters before and after processing revealed that both resulted in decreased sperm concentration after processing (*P*=0.000006 for sperm washing followed by swim-up; and *P*=0.008457 for discontinuous density gradient centrifugation), and increased motility and rates of morphologically normal sperm *(P*=0.00001 for sperm washing followed by swim-up; and *P*<0.05 for discontinuous density gradient centrifugation) after processing (Tables 1 and 2).

**Table 1:** Comparison of seminal parameters before and after semen processing through sperm washing followed by swim-up.

Sperm washing followed by swim up (n=17)	Before semen processing Mean ± Std Dev	After semen processing Mean ± Std Dev	*P*-value *
**Concentration (x10^6^/ml)**	92.5 ± 56.9	29.0 ± 28.2	0.000006
**Motility (%)**	52.8 ± 12.9	76.5 ± 18.5	0.000001
**Morphology (%)** normal abnormal	29.8 ± 7.0 70.1 ± 7.0	56.7 ± 12.8 43.2 ± 12.8	0.000001 0.000001

Std Dev = standard deviation

* Dependent Student’s t-test with significant difference; *P* < 0.05

**Table 2:** Comparison of seminal parameters analyzed before and after semen processing through discontinuous density gradient centrifugation.

Isolate (n=41)	Before semen processing Mean ± Std Dev	After semen processing Mean ± Std Dev	*P*- value *
**Concentration (x10^6^/ml)**	81.1 ± 52.6	64.8 ± 43.3	0.008457
**Motility (%)**	58.0 ± 17.7	76.0 ± 17.4	0.000000
**Morphology (%)** normal abnormal	29.6 ± 11.2 70.3 ± 11.2	45.3 ± 15.2 54.0 ± 14.6	0.000000 0.000000

Std Dev=standard deviation

* Dependent Student’s t-test with significant difference; *P* < 0.05

However, when the results before processing of both techniques were compared, no statistically significant differences were found in regards to sperm concentration, motility, or morphology (Table 3).

**Table 3:** Comparison of the results of sperm washing followed by swim-up and discontinuous density gradient centrifugation before and after semen processing.

		Sperm washing followed by swim-up (n=17) Mean ± Std Dev	Isolate (n=41) Mean ± Std Dev	*P*-value *
**Concentration** **(x10^6^/ml)**	**before**	92.5 ± 56.9	81.1 ± 52.6	0.4642
	**after**	29.0 ± 28.2	64.8 ± 43.3	0.0027
**Motility (%)**	**before**	52.8 ± 12.9	58.0 ± 17.7	0.2748
	**after**	76.5 ± 18.5	76.0 ± 17.4	0.9285
**Morphology (%)** normal				
	**before**	29.8 ± 7.0	29.6 ± 11.2	0.9450
	**after**	56.7 ± 12.8	45,3 ± 15.2	0.0095
**abnormal**	**before**	70.1 ± 7.0	70.3 ± 11.2	0.9450
	**after**	43.2 ± 12.8	54.0 ± 14.6	0.0106

Std Dev=standard deviation

* Independent Student’s t-test with significant difference; *P* < 0.05

Unlike motility (*P*=0.9825), the differences in semen concentration (*P*=0.0027) and morphology (normal *P*=0.0095; abnormal *P*=0.0106) between the two techniques were statistically significant. In other words, the sperm concentration recovery rates seen in discontinuous density gradient centrifugation (64.8 ± 43.3) were higher than the rates observed in sperm washing followed by swim-up (29.0 ± 28.2) (*P*=0.0027), whereas morphology recovery rates were better in sperm washing followed by swim-up (56.7 ± 12.8) than in discontinuous density gradient centrifugation (45.3 ± 15.2) (*P*=0.0095) (Table 3).

### DISCUSSION

Swim-up and discontinuous density gradient are the most commonly used semen preparation techniques in AHR laboratories (Paasch *et al*., 2007; Jayaraman *et al*., 2012).

When used in IUI, these techniques are expected to provide at least five million motile spermatozoa. This number of sperm cells is required so that fertilization may occur in the fallopian tube. Concentrations below this limit suggest the choice of other AHR methods such as in-vitro fertilization (IVF) (Khalil *et al*., 2001; Wainer *et al*., 2004). The choice of method is based on the baseline quality of the semen sample. According to the WHO (WHO, 1999), either technique can be used for normal semen (Wainer *et al*., 2004).

Few studies in the literature have compared the swim-up and discontinuous density gradient techniques, and different conclusions have been proposed about them (Carrell *et al*., 1998; Dodson *et al*., 1998; Posada *et al*., 2005). In a meta-analysis investigating different semen preparation techniques used for IUI, the results were qualitatively and quantitatively inconclusive, as the data were insufficient to conclude which technique was superior (Boomsma *et al*. 2007).

Both sperm capacitation methods used in the present study recovered the minimum amounts of sperm required to perform IUI. Our results agree with the results presented by Khalil *et al*. (2001). The comparison of the two techniques before sperm preparation revealed that none was favored.

After sperm preparation, lower sperm concentration recovery rates and higher morphology recovery rates were observed in sperm washing followed by swim-up than in discontinuous density gradient centrifugation. These findings indicate an increase in sperm selection wherein concentration decreases as morphologically normal spermatozoa are selected, as seen when the two methods were compared for sperm morphology after preparation, revealing that sperm washing followed by swim-up had better results in normal sperm recovery rates than discontinuous density gradient centrifugation (*P*=0.0095). Our results are in agreement with the results reported by Fraczek *et al*. (2004), which showed that sperm selected by swim-up presented slightly better viability and morphology than cells isolated by discontinuous density gradient centrifugation. In contrast, Prakash *et al*. (1998) and Hammadeh *et al*. (2001) found a higher percentage of morphologically normal sperm after using discontinuous density gradient centrifugation. Xue *et al.* (2014) compared swim-up and discontinuous density gradient centrifugation in patients with teratozoospermia, and their results suggested that enrichment with normal morphology spermatozoa with intact DNA could be achieved through swim-up or discontinuous density gradient centrifugation, when compared to unprocessed semen. Other authors have reported similar results (Ng *et al*., 1992; Hammadeh *et al*., 2001; Chiamchanya *et al*., 2010). Furthermore, Xue *et al*. (2014) also observed that swim-up produced a higher sperm deformity rate than discontinuous density gradient centrifugation, which produced more favorable results. These findings echo with the data reported by Hammadeh *et al*. (2001) and Jayaraman *et al*. (2012), who described a higher percentage of morphologically normal spermatozoa using discontinuous density gradient centrifugation in infertile and teratozoospermic patients, respectively. However, Borges *et al.* (2013) found no significant differences in the percentages of morphologically normal sperm cells between the two methods in infertile patients. This may be explained by differences in the centrifugation medium used or the type of patients selected in each study.

In the present study, when we compared the results from the two techniques after semen processing, discontinuous density gradient centrifugation had better concentration recovery rates than sperm washing followed by swim-up; our results concord with Huang *et al*. (1996). Sperm concentrations below 5x10^6^ have been associated with low counts of sperm with normal morphology, and impaired sperm motility in such cases is suggestive of lower chances of fertilization (Wainer *et al*., 2004). According to Karamahmutoglu *et al*. (2014), discontinuous density gradient centrifugation significantly improves clinical outcomes (pregnancy rates) in IUI cycles of couples with unexplained subfertility and favorable seminal parameters when compared to the swim-up technique. The two techniques produce similar clinical outcomes for subfertile men. This confirms that the efficacy of discontinuous density gradient centrifugation is more pronounced in couples with unexplained subfertility, in which seminal parameters are within the normal range. In addition, Karamahmutoglu *et al*. (2014), showed that, contrary to their own results, in situations where neither method was superior in male factor patients, it has been accepted that discontinuous density gradient centrifugation is the preferred method in IUI cycles in which sperm counts are low or sperm motility is impaired. Despite evidence suggesting increased pregnancy rates with discontinuous density gradient centrifugation, the authors concluded that their results did not reveal the evidence to support a possible explanatory mechanism.

Motility increased significantly and similarly with both techniques when they were analyzed separately; when compared to each other, no statistically significant differences were found. According to Huang *et al*. (1996), discontinuous density gradient centrifugation allows for greater recovery of highly motile sperm to perform IUI. However, Posada *et al.* (2005) showed that the increased clinical pregnancy rates associated with the use of swim-up could be explained by a significantly greater number of totally motile spermatozoa in pre-wash and post-wash semen samples, when compared to discontinuous density gradient centrifugation. According to some authors, recovery rates of totally motile, progressively motile, and viable sperm cells were higher after with discontinuous density gradient centrifugation than with swim-up (Ding *et al*., 2000; Ricci *et al.*, 2009). However, Chantler *et al*. (2004) observed that the ratio of fast sperm was enhanced with the swim-up method.

Another point to be considered is the presence of leukocytes that produce reactive oxygen species (ROS), which change the sperm membrane and might cause sperm DNA fragmentation (Aitken & Clarkson, 1987; Aitken & Clarkson, 1988; Griveau & Lannou, 1994; Aitken *et al*., 1995). According to Li *et al*. (2012), the two techniques produce decreased rates of sperm DNA fragmentation after semen processing, while other studies have shown that discontinuous density gradient centrifugation efficiently selects sperm with better DNA and chromatin structures (i.e., sperm with greater fertilization potential) (Sakkas *et al*., 2000; Morrell, 2004).

During the washing procedures, sperm and other components present in the ejaculate settle down, excluding only the seminal plasma, which increases the contact of spermatozoa with other cells that produce ROS during the incubation period for the swim-up technique (Ford, 1990). The discontinuous density gradient centrifugation method already mechanically separates leukocytes, debris, and most of the dead sperm cells. The highly functional sperm selected are exposed to cells that produce ROS for a shorter time period in discontinuous density gradient centrifugation than in swim-up (Jayaraman *et al.*, 2012). Sperm recovery by discontinuous density gradient centrifugation separates motile sperm from other cells, and is a good alternative for cases with higher concentrations of leukocytes, debris, and dead sperm.

Both techniques have advantages and disadvantages. Sperm washing followed by swim-up is an inexpensive technique in laboratory settings, and takes approximately twice as long as discontinuous density gradient centrifugation; furthermore, while the samples are incubated the lab technician can perform other tasks in the laboratory. When using the swim-up technique, sperm quality (i.e., semen with higher sperm concentration) should be taken into account (Henkel & Schill, 2003). However, this method presents some disadvantages: it is restricted to ejaculates with high sperm count and motility; it is a low-yield technique; spermatozoa can be massively damaged by ROS; and there is a significant decrease in the percentage of normally chromatin-condensed spermatozoa (Henkel & Schill, 2003). The swim-up is a more sensitive technique, in that it has a greater number of factors that may influence the results. Technicians should be specially trained on this technique, so that the final result is the best possible (Neves, 1991).

Discontinuous density gradient centrifugation is easy to perform and yields higher sperm concentrations with less preparation time (approximately 55 minutes). It also has fewer critical points sensitive to error during preparation, as well as higher levels of effectiveness. However, during discontinuous density gradient centrifugation the technician stays in the lab for longer uninterrupted stretches of time from the beginning to the end of the procedure, thus preventing him/her to perform other tasks in the laboratory. According to Henkel & Schill (2003), the disadvantages are that the production of good interphases between the different media is a little more time-consuming, and a bit more expensive.

Another important factor relates to the costs of the two techniques. A better-equipped and prepared laboratory is required to perform sperm washing followed by swim-up, when compared to discontinuous density gradient centrifugation. This can be an important factor when choosing a semen processing technique. If one chooses to consider only the cost of the materials used during each method, discontinuous density gradient centrifugation is more expensive, as also observed by Neves (1991).

### CONCLUSION

In conclusion, discontinuous density gradient centrifugation presents good semen concentration, motility, and morphology recovery rates after processing; it is a great option for individuals who do not have high sperm concentration; it is less effective in morphological selection; and produces higher concentration rates than sperm washing followed by swim-up. Sperm washing followed by swim-up is indicated for semen with high sperm concentration levels, because it presents a lower concentration recovery rate than discontinuous density gradient centrifugation; however, it does provide for more efficient morphological selection, mainly for IVF (Englert *et al*., 1992; Sapienza *et al*., 1993). The main limitations of the present study revolve around the small number of patients and the fact that all semen samples were normospermic.
